# The prognostic role of circulating tumour DNA detected prior to clinical diagnosis of colorectal cancer in the HUNT study

**DOI:** 10.1186/s12885-024-13030-x

**Published:** 2024-10-10

**Authors:** Siv Stakset Brenne, Poul Henning Madsen, Inge Søkilde Pedersen, Kristian Hveem, Frank Skorpen, Henrik Bygum Krarup, Athanasios Xanthoulis, Eivor Alette Laugsand

**Affiliations:** 1 Department of Surgery, Levanger Hospital, Nord-Trøndelag Hospital Trust, Levanger, Norway; 2 Department of Public Health and Nursing, HUNT Research Centre, Norwegian University of Science and Technology, Levanger, Norway; 3 Clinical Cancer Research Centre, Aalborg University Hospital, Aalborg, Denmark; 4 Molecular Diagnostics, Aalborg University Hospital, Aalborg, Denmark; 5 Department of Clinical Medicine, Aalborg University, Aalborg, Denmark; 6 Department of Clinical and Molecular Medicine, Norwegian University of Science and Technology, NTNU, Trondheim, N-7489 Norway

**Keywords:** Circulating tumour DNA, DNA methylation, Liquid biopsy, Colorectal cancer, Prognosis

## Abstract

**Background:**

Today, the prognostic tools available at the time of diagnosis in colorectal cancer (CRC) are limited. Better prognostic tools are a prerequisite for personalised treatment.

This study aimed to investigate whether circulating tumour DNA (ctDNA) markers found in plasma before clinical diagnosis of CRC could contribute to the prediction of poor prognosis.

**Methods:**

This observational cohort study included patients diagnosed with CRC stage I-III within 24 months following participation in the Trøndelag Health Study (*n* = 85). Known methylated ctDNA biomarkers of CRC were analysed by PCR in plasma. Outcomes were overall survival (OS), recurrence-free survival (RFS) and poor prognosis (PP). Candidate clinical and methylated ctDNA predictors of the outcomes were identified by Cox regression analyses.

**Results:**

Methylated *GRIA4* (HR 1.96 (1.06–3.63)), *RARB* (HR 9.48 (3.00–30.00)), *SLC8A1* (HR 1.97 (1.03–3.77)), *VIM* (HR 2.95 (1.22–7.14)) and *WNT5A* (HR 5.83 (2.33–14.56)) were independent predictors of OS, methylated *RARB* (HR 9.67 (2.54–36.81)), *SDC2* (HR 3.38 (1.07–10.66)), *SLC8A1* (HR 2.93 (1.01–8.51)) and *WNT5A* (HR 6.95 (1.81–26.68)) were independent predictors of RFS and methylated *RARB* (HR 6.11 (1.69–22.18)), *SDC2* (HR 2.79 (1.20–6.49)) and *WNT5A* (HR 5.57 (3.04–15.26)) were independent predictors of PP (*p* < 0.05).

**Conclusions:**

Prediagnostic ctDNA markers are promising contributors to predicting poor prognosis in CRC, potentially becoming one of the tools guiding more personalised treatment.

**Supplementary Information:**

The online version contains supplementary material available at 10.1186/s12885-024-13030-x.

## Introduction

Worldwide, colorectal cancer (CRC) is a frequent malignancy, inflicting high social and individual costs [1]. Diagnosis, treatment, and follow-up of the disease is resource demanding. Although most patients are diagnosed at early, curable stages (stage I-III 65%) [2], cancer recurs in up to 40% of patients [3]. Five-year survival ranges from over 90% in stage I CRC to less than 10% in stage IV CRC, and survival within each stage varies widely [4]. Precise prediction of prognosis is a key factor in clinical decision-making and communication with patients. Today, the primary tools to guide treatment choice and predict prognosis are stage, histological characteristics, age and comorbidity.

The diagnosis and clinical TNM-stage (cTNM) in CRC are based on tissue biopsies, clinical and radiologic findings. cTNM guides the choice of treatment and is extensively used as a prognostic indicator. However, adjustment of the TNM-stage (pTNM or ypTNM) after histologic assessment of a surgical specimen is common (cTNM correct in 28% and 19% of rectal and colon cancers, respectively) [5], and locoregional over- and understaging based on CT-scans resulted in inappropriate treatment strategies in up to 48% of patients [6, 7].

In addition to TNM, established findings relevant for the prediction of recurrence and survival are tumour differentiation grade, perioperative bowel obstruction or perforation, the presence of lymphovascular invasion (LVI), the number of investigated lymph nodes (< 12 vs ≥ 12), extramural vascular invasion (EMVI), perineural invasion (PNI), high tumour budding, compromised surgical margins (R1 resection with margin ≤ 1 mm in any direction), age and comorbidities. Genetic factors such as mismatch repair (MMR) deficiency, microsatellite instability (MSI), *RAS/RAF* mutations, *HER2* amplification and *NTRK* gene fusions are also important [8, 9]. As exemplified for high-risk stage II colon cancer, the presence of one or more of these postsurgical risk factors strongly predicts disease recurrence [4].

With new advances in colorectal cancer treatment (i.e., immunotherapy and more radical surgeries), it is evident that prediction models in CRC can no longer be based exclusively on variables available after surgery. Circulating tumour DNA (ctDNA) has emerged as a promising prognostic biomarker in CRC [10]. By being a liquid biopsy, ctDNA may provide a more complete picture of the tumour heterogeneity [11]. In metastatic CRC, 80–90% of patients have detectable ctDNA, and higher levels of ctDNA are associated with higher tumour burden, liver metastases, shorter progression-free survival (PFS) and overall survival (OS) [12]. In nonmetastatic CRC, methylated ctDNA is less studied, but several studies have found ctDNA markers to have predictive value for both recurrence and survival [13].

In CRC, more nuanced prognostic tools are needed to avoid unnecessary side effects, over- and undertreatment, and would preferably be based on data available from the diagnostic workup of the patients. Screening, and possibly even earlier detection of CRC by ctDNA-based liquid biopsies [14], may move the time of diagnosis upstream. This study aimed to determine whether known ctDNA markers found in plasma before clinical diagnosis of CRC can contribute to the prediction of poor prognosis.

## Materials and methods

### Study design

This prospective cohort study is based on the third wave of the cross-sectional Trøndelag Health Study (HUNT3 2006–2008). The adult population ≥ 20 years old in Nord-Trøndelag County was invited, where approximately 60 000 participated [15]. HUNT3 included written questionnaires, clinical measurements and blood samples. Through linkage between HUNT and the Cancer Registry of Norway (CRN), we included subjects diagnosed with colorectal adenocarcinoma stage I-III in the CRN ≤ 24 months after participation in HUNT3. Colorectal adenocarcinoma was defined by the International Classification of Diseases, 10th edition [ICD-10]: C18-20 (excluding C18.1 Appendix [ICD-7 code 153.6]); International Classification of Diseases for Oncology, 3rd edition [ICD-O-3]: 8140, 8144, 8210, 8211, 8255–8263, 8480–8481, 8490, 8510, 8570–8574, 6900, 6999, 8000–8020 (excluding 8041,8240–8246, 8249 and 8936). The course of their disease in terms of death or recurrence was followed until July 2018. No exclusion criteria were applied, including previous cancer history.

### Outcome and predictor variables

The outcome variables of this study were overall survival (OS), recurrence-free survival (RFS) and poor prognosis (PP). OS and RFS were defined according to the DATECAN initiative [16]. For RFS analyses, cases that never were cancer free were excluded. As a clinically useful endpoint, poor prognosis (PP) was defined as recurrence at any time or death within 5 years following primary treatment. The dates of finishing primary treatment and recurrence were found in the medical records, whereas the date of diagnosis was provided by the CRN. Dates of death or loss to follow-up were registered in the HUNT study. Based on previous research, methylated ctDNA biomarkers within 21 genes were identified as potential prognostic variables and handled as dichotomous (methylated/nonmethylated) [17–22]. For selection of panels of ctDNA markers for the 3 outcomes, the ctDNA markers with best ability to separate the specific outcomes were chosen (*p* < 0.05). Information about sex, age, smoking and body mass index (BMI) was extracted from HUNT data. Carcinoembryonic antigen (CEA) level, haemoglobin (Hb) level, stage according to the American Joint Committee on Cancer staging system 7th Edition (AJCC-stage I-IV) [23], TNM-stage [24] and treatment were extracted from the medical records. Standard treatment of care in Norway includes formal surgical resection for stage I-II CRC, preoperative radio chemotherapy for high risk rectal cancer (T4, ≤ 2 mm margin from tumour or tumour deposits to the mesorectal fascia (MRF), ≤ 1 mm to pathological lymph node, extramural vein invasion, N1 outside the MRF, low tumours with short expected circumferential resection margins) and adjuvant chemotherapy for stage III colon cancer.

### Blood samples

All blood samples were obtained by a skilled technician, transported to HUNT Research Centre at 4 °C, centrifuged at 6 °C for 10 min at 2500 g and aliquoted within 24 h after venipuncture. The EDTA plasma aliquots were stored at -80 °C. To blind the assay operators of phenotype, all frozen samples were given a unique ID-number before being couriered to Aalborg University Hospital for the methylation analyses.

### Analyses of methylated promoter regions in ctDNA

Plasma nucleic acids were extracted from 2 × 900 μl EDTA plasma according to the manufacturer’s instructions, using the easyMAG™ platform (NucliSens® [bioMerieux SA, France]). Purified nucleic acids were eluted in 2 × 25 μl elution buffer. For quantitation of extracted DNA 5 μl was used, and the remainder was deaminated for 10 min at 90 °C by mixing with 90 μl of deamination solution, followed by purification using EasyMag and elution in 25 μl of 10 mM KOH. Within each gene of interest, primers and probes were designed to target two markers (amplicon sizes and detailed PCR descriptions in Tables S1 and S2). DNA extraction and methylation analysis were based on a rapid bisulfite-treatment of cell-free DNA extracted from plasma samples with a 2-step PCR detection, as described in a previously published protocol [25].

### Ethics

All participants in HUNT gave written informed consent, including consent for connection to their medical records and other central health registries in Norway. In addition, specific ethical approval for this particular study was obtained from the Regional Committee for Medical and Health Research Ethics. All data were stored and handled confidentially, and only the research team had access.

### Statistical analyses

The following possible predictor variables were handled as dichotomous: sex (male or female (reference)), age (> 80 or ≤ 80 years (reference)), smoking (> 20 or ≤ 20 pack years (reference)), BMI (> 25 or ≤ 25 kg/m^2^ (reference)), Hb (< 13.4 or ≥ 13.4 g/dl (reference) for male, < 11.7 or ≥ 11.7 g/dl (reference) for female), CEA (≥ 5 or < 5 μg/l (reference)), and tumour location (colon or rectum (reference)). The reference category was assigned so that the association between the predictor variable and the primary outcome was positive. For missing values (*n* = 7 for CEA) the median value 3.0 was imputed and for Hb the mean value 12.8 was imputed (*n* = 3 males). There were no other missing data.

For comparisons between groups, the chi-square test was used for categorical variables and the independent samples t-test was used for continuous variables. Survival analyses were performed using Cox regression analyses and Kaplan–Meier plots, comparing the survival distributions by the log-rank test. First, Cox regression analyses adjusted for age were performed for the ctDNA markers (Table S3). Second, markers that were independent predictors (*p* < 0.05) of the outcomes in the adjusted analyses, were combined into panels (HUNT-CRC_OS/RFS/PP_). For the number of markers present in each panel and other potential predictors of survival, hazard ratios were estimated by univariable and multivariable Cox regression survival analysis. All analyses were performed in SPSS® version 28 (IBM®), and the predefined significance level was set to 0.05.

## Results

### Characteristics of the study population

In the 24 months following HUNT3, 85 participants were diagnosed with stage I-III CRC and included in this study (Table 1). All of the 85 included cancer patients underwent formal surgical resection with curative intent. We had information on the entire treatment course for 79% of the included patients, and all received standard treatment of care for Norway at the time. There were 46 females and 39 males in the cohort, with a mean age of 70.0 years (Table 1). Nine percent of cases were stage I, 60% stage II and 30% stage III (Table 1). The mean BMI was 27.3, the mean CEA level at diagnosis was 8.6 and the mean number of pack-years smoked was 14.4 (Table 1). Six percent of participants had diabetes mellitus, and 20% had had other cancers during their lifetime (65% before participation (range 3–35 years before) and 35% after participation (range 2–9 years after)) (Table 1). Seventy-three percent of the cancers were located in the colon, whereas 27% were located in the rectum (Table 1). During the observation period, 42 patients died, 14 had recurrence, and there were 25 outcomes of poor prognosis (Table 1). There were no recurrences more than 43 months after primary treatment and no patients were lost to follow-up (Table 1). The mean time from HUNT3 participation to diagnosis was 12.2 months (Table 1). The occurrence of each methylated marker in the cohort is described in Table S3.

**Table 1:** Characteristics of the patients with stage I-III CRC

	**Cohort**	**Missing**
Total, n	85	n
Sex, n (%)		
Male	39 (45.9)	0
Female	46 (54.1)	0
Age, mean (SD)	70.0 (10.1)	0
BMI, mean (SD)	27.3 (4.2)	0
Smoking, mean (SD)	14.4 (16.4)	0
Hb, mean (SD)		3
Male	12.8 (2.4)	
Female	11.7 (1.8)	
CEA, mean (SD)	8.6 (25.2)	7
Diabetes, n (%)	5 (5.9)	0
Other cancers, n (%)	17 (20.0)	0
Tumour localisation		0
Colon	62 (72.9)	
Rectum	23 (27.1)	
AJCC stage, n (%)		0
I	8 (9.4)	
II	52 (61.2)	
III	25 (29.4)	
Deaths, n (%)	42 (49.4)	0
Recurrences, n (%)	14 (16.5)	0
PP events^a^, n (%)	25 (29.4)	0
HUNT3 to diagnosis, mean (SD)	12.2 (6.9)	0
Lost to follow up, n (%)	0 (0)	0

*SD* Standard deviation, *AJCC* American Joint Committee on Cancer, *n* number

^a^recurrence or death < 5 years following diagnosis, OS, overall survival in months, RFS, recurrence-free survival in months, HUNT3 to diagnosis in months, smoking in pack years, BMI, body mass index in kg/m^2^, age in years, CEA, carcinoembryonic antigen in µg/l, PP, poor prognosis, CRC, colorectal cancer

### Overall survival and recurrence-free survival

Methylated *GRIA4* (HR 1.96 (1.06–3.63)), *RARB* (HR 9.48 (3.00–29.98)), *SLC8A1* (HR 1.97 (1.03–3.77)), *VIM* (HR 2.95 (1.22–7.14)) and *WNT5A* (HR 5.83 (2.33–14.56)) were independent markers of OS (*p* < 0.05). For RFS, methylated *RARB* (HR 9.67 (2.54–36.81)), *SDC2* (HR 3.38 (1.07–10.66)), *SLC8A1* (HR 2.93 (1.01–8.51)) and *WNT5A* (HR 6.95 (1.81–26.68)) were independent markers (*p* < 0.05) (Table S3).

### Poor prognosis

Methylated *RARB* (HR 6.11 (1.69–22.18)), *SDC2* (HR 2.76 (1.20–6.49)) and *WNT5A* (HR 5.57 (3.04–15.26)) were independent markers for PP (*p* < 0.05) (Table S3).

### HUNT-CRC prognostic panels

**Fig. 1:**
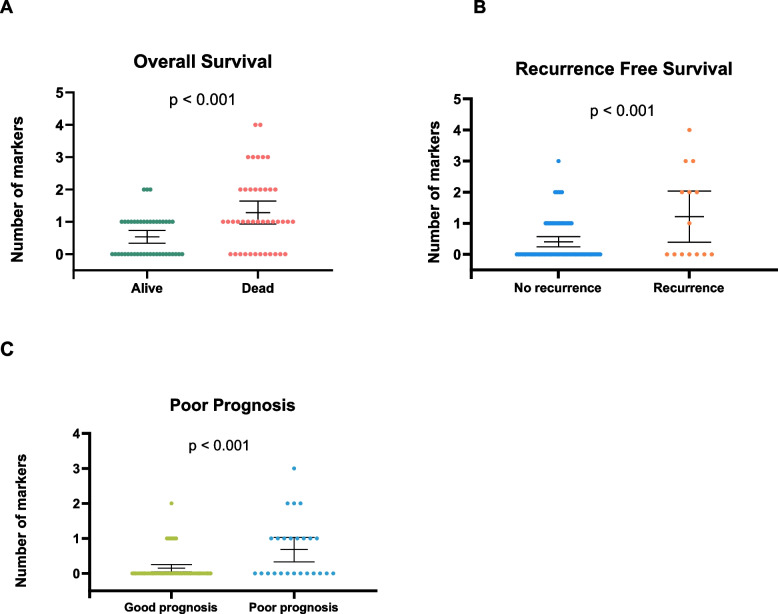
Number of markers in OS, RFS and PP

**Fig. 2:**
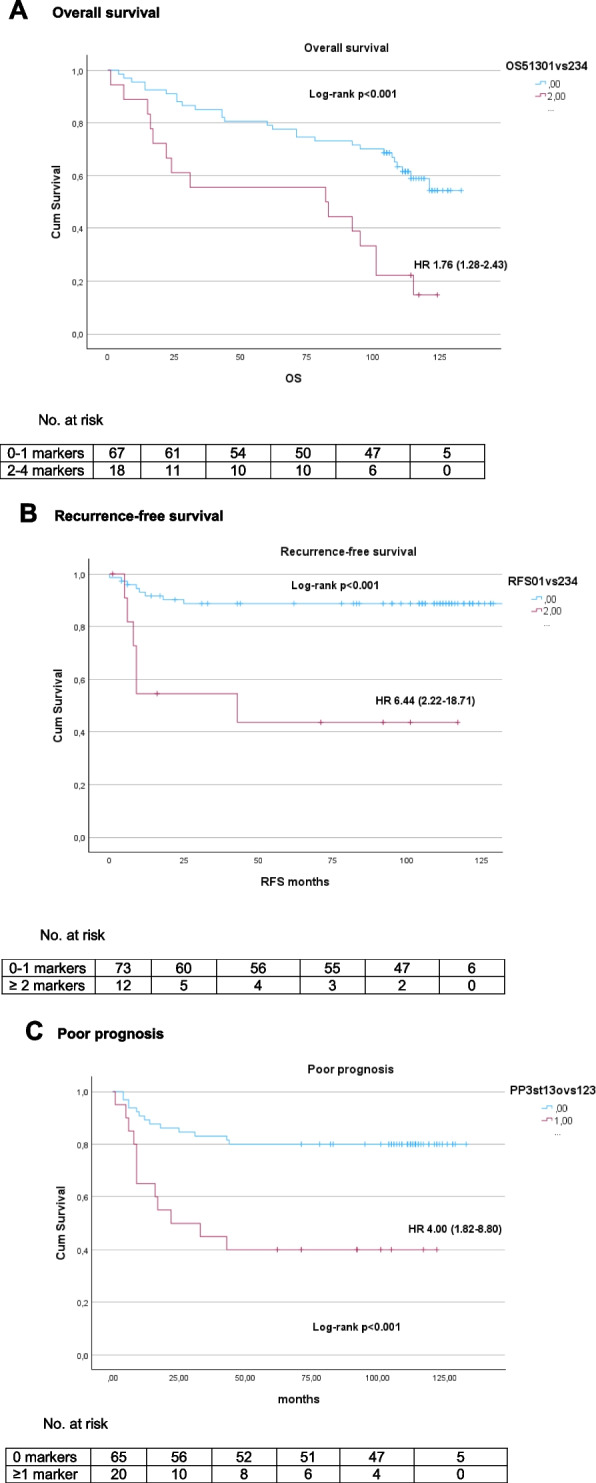
Kaplan Meier curves by number of markers in OS, RFS and PP

When combining the independent markers above for the three different outcomes into panels (*GRIA4*, *RARB*, *SLC8A1*, *VIM* and *WNT5A* = HUNT-CRC_OS_ for OS, *RARB*, *SDC2*, *SLC8A1* and *WNT5A* = HUNT-CRC_RFS_ for RFS and *RARB*, *SDC2* and *WNT5A* = HUNT-CRC_PP_ for PP), the number of markers in the panels was significantly associated with each outcome (*p* < 0.001) (Fig. 1). As illustrated in Fig. 2, having two or more of the markers in each panel was significantly associated with OS (HR 1.76 (1.26–2.43), *p* < 0.001) and RFS (HR 6.44 (2.22–18.71), *p* < 0.001). Having 1 or more of the markers in HUNT-CRC_PP_ was significantly associated with PP (HR 4.00 (1.82–8.80), *p* < 0.001). For cases with ≥ 2 markers, 40% died within the first 2 years, compared to cases with ≤ 1 marker, where approximately 90% were alive (Fig. 2A). Within the group with ≥ 2 markers, 50% had recurrence within the first 10 months, whereas only 4% of those with ≤ 1 marker had recurrence in the same period (Fig. 2B). For the cases with ≥ 1 marker 60% had recurrence or died within the first four years, while 90% of those with no markers were recurrence free and alive after the same time period (Fig. 2C). Comparisons between the two groups within OS, RFS and PP were made, and there were no significant differences other than tumour localisation and AJCC stage in RFS and age and smoking in PP (Table S4).

In univariate and multivariate analyses, the number of markers in the HUNT-CRC_OS/RFS/PP_ panels was an independent predictor for OS, RFS and PP (Tables 2, 3 and 4). For OS, having ≥ 2 markers was an independent predictor (HR 1.66 (1.26–2.19), *p* < 0.001) as was age > 80 years (HR 3.01 (1.52–5.99), *p* = 0.002) (Table 2). For RFS, having ≥ 2 markers (HR 3.86 (1.18–12.68), *p* = 0.026) was an independent predictor (Table 3). Having ≥ 1 marker (HR 3.40 (1.46–7.93), *p* = 0.005) and CEA > 5 µg/l (HR 2.92 (1.28–6.66), *p* = 0.011) were independent predictors of PP (Table 4). Notably, AJCC stage was not an independent predictor of OS; RFS or PP (Tables 2, 3 and 4).

**Table 2:** Cox regression analyses of OS in stage I-III CRC patients

	**Univariable**		**Multivariable**	
	**HR (95% CI)**	***p*-value**	**HR (95% CI)**	***p*-value**
HUNT-CRC_OS_ (≥ 2 marker)	1.75 (1.34–2.29)	** < 0.001**	1.63 (1.14–2.35)	**0.008**
Male sex	1.23 (0.67–2.25)	0.505		
Age > 80 years	3.63 (1.84–7.17)	** < 0.001**	3.03 (1.51–6.08)	**0.002**
Smoking > 20 pack years	1.14 (0.57–2.26)	0.719		
BMI > 25 kg/m^2^	0.81 (0.42–1.53)	0.507		
Hb < 13.4 Male/ < 11.7 Female	1.70 (0.90–3.22)	0.101		
CEA > 5 μg/l	1.46 (0.73–2.89)	0.282		
Location colon	1.28 (0.63–2.60)	0.502		
AJCC stage	1.37 (0.82–2.29)	0.227	0.98 (0.56–1.71)	0.978

*AJCC* American Joint Committee on Cancer, *OS* Overall survival in months, smoking in pack years, *BMI* Body mass index in kg/m^2^, age in years, *CEA* Carcinoembryonic antigen in µg/l, *HR* Hazard ratio, *CI* Confidence interval, *CRC* Colorectal cancer

**Table 3:** Cox regression analyses of RFS in stage I-III CRC patients

	**Univariable**		Multivariable	
	**HR (95% CI)**	***p*-value**	**HR (95% CI)**	***p*-value**
HUNT-CRC_RFS_ (≥ 2 markers)	6.44 (2.22–18.71)	** < 0.001**	3.86 (1.18–12.68)	**0.026**
Male sex	2.89 (0.91–9.23)	0.073		
Age > 80 years	1.02 (0.23–4.58)	0.976		
Smoking > 20 pack years	0.75 (0.21–2.71)	0.665		
BMI > 25 kg/m^2^	0.73 (0.25–2.19)	0.576		
Hb < 13·4 Male/ < 11·7 Female	1.83 (0.61–5.46)	0.279		
CEA > 5 μg/l	3.49 (1.21–10.07)	**0.021**	2.72 (0.91–8.12)	0.072
Location colon	0.62 (0.21–1.84)	0.384		
AJCC stage	2.78 (1.08–7.19)	**0.035**	1.68 (0.58–4.85)	0.338

*AJCC* American Joint Committee on Cancer, *RFS* Recurrence-free survival in months, HUNT3 to diagnosis in months, smoking in pack years, *BMI* Body mass index in kg/m^2^, age in years, *CEA* Carcinoembryonic antigen in µg/l, *HR* Hazard ratio, *CI* Confidence interval, *CRC* Colorectal cancer

**Table 4:** Cox regression analyses of PP in stage I-III CRC patients

	**Univariable**		**Multivariable**	
	**HR (95% CI)**	*p***-value**	**HR (95% CI)**	***p*-value**
HUNT-CRC_PP_ (≥ 1 marker)	4.00 (1.82–8.80)	** < 0.001**	3.03 (1.24–7.41)	**0.015**
Male sex	0.93 (0.42–2.06)	0.866		
Age > 80 years	2.99 (1.29–6.95)	**0.011**	1.72 (0.69–4.30)	0.249
Smoking > 20 pack years	0.72 (0.27–1.91)	0.508		
BMI > 25 kg/m^2^	0.61 (0.28–1.36)	0.229		
Hb < 13·4 Male/ < 11·7 Female	1.43 (0.64–3.17)	0.385		
CEA > 5 μg/l	3.05 (1.34–6.93)	**0.008**	2.81 (1.22–6.43)	**0.015**
Location colon	0.93 (0.39–2.22)	0.868		
AJCC stage	1.82 (0.92–3.59)	0.084	1.35 (0.62–2.91)	0.451

*AJCC* American Joint Committee on Cancer, HUNT3 to diagnosis in months, smoking in pack years, *BMI* Body mass index in kg/m^2^, age in years, *CEA* Carcinoembryonic antigen in µg/l, *PP* Poor prognosis, *CRC* Colorectal cancer, *HR* Hazard ratio, *CI* Confidence interval

## Discussion

By analysing six methylated ctDNA biomarkers, we were able to identify CRC patients with worse OS and RFS, as well as a high risk of recurrence or death within 5 years (PP). To our knowledge, this is the first study demonstrating a promising value of ctDNA as a prognostic biomarker found in plasma even up to two years prior to the clinical diagnosis of CRC. These are important findings, as the field of CRC needs new effective prognostic biomarkers preferably available to clinicians upon diagnosis.

The studied population has a high incidence of CRC, and many are diagnosed in advanced stages [2], partly because a national screening programme is not fully implemented in Norway to date. CRC screening has been implemented in recent years in most developed countries, resulting in reduced incidence and downstaging of the disease [26]. Unfortunately, most screening programmes have low participation rates (approximately 50%) [26], primarily due to hesitancy towards faecal sample collection or endoscopic examinations. Minimally invasive tests such as a blood sample could improve screening participation [27]. As previously shown by our research group, methylated ctDNA markers could detect CRC up to two years prior to a clinical diagnosis [14]. Hence, the timing of blood sampling in this study could represent a future point of diagnosis, possibly allowing more personalised treatment and hopefully reducing over- and undertreatment.

Consistent with previous studies, we found that methylated *GRIA4, RARB, VIM* and *WNT5A* were independently associated with CRC prognosis. Methylated *GRIA4* has value in monitoring of treatment response and has predictive and prognostic value in metastatic CRC disease [28, 29]. Additionally, methylated *GRIA4* has promising value as a marker of metastatic disease in CRC [30]. This study expands the potential use of *GRIA4* as a prognostic biomarker also in non-metastatic CRC disease. Methylated *RARB* has been associated with overall survival and prognosis in all stage CRC [31] and other cancers [32, 33]. Methylated *VIM* has been associated with poor prognosis and advanced stage in CRC [34], as well as for survival [35] and recurrence [36] in other cancers. *WNT5A* is associated with a better response and longer PFS in patients treated with 5-fluorouracil (5-FU) in CRC cell cultures [37]. Additionally, *WNT5A* is associated with prognosis in several other cancers [38]. This study adds potential value of methylated *WNT5A* as a marker of prognosis in non-metastatic CRC.

To our knowledge, no major studies have investigated methylated *SDC2* and *SLC8A1* as prognostic biomarkers in CRC. *SDC2* has been extensively investigated as a diagnostic biomarker of CRC [34]. In vitro studies have shown that *SDC2* contributes to 5-FU resistance in CRC cells, as well as to increased proliferation, migration and invasion, and inhibition of apoptosis [39]. The *SLC8A1* gene is involved in calcium reabsorption and homeostasis, which regulates proliferation and apoptosis in many cells, including colon cancer cells [40]. The above mentioned functions of the genes could potentially explain the prognostic significance when altered. Although little is known about the prognostic role of *SLC8A1* in CRC, potential is implied in several other cancers [41, 42].

Although other studies have combined panels of methylated biomarkers [3, 29, 43, 44], we believe this is the first study demonstrating a prognostic value of combining methylated ctDNA markers available upstream of clinical diagnosis.

There are several possible limitations of this study. First, the blood samples were drawn up to 24 months before clinical diagnosis and staging, hence the stage may have been an earlier one at the time of blood sampling than at the time of clinical diagnosis. This makes the amount of ctDNA smaller and harder to detect. However, as colorectal cancer takes years to develop and methylation is an early event in the carcinogenesis, most cases probably had the disease at the time of blood-sampling. The abovementioned facts could make the findings of the results more modest than in real-life. In the future, screening could shift the time of diagnosis upstream, so that the timing of diagnosis could be closer to the plasma sampling time in the present study.

Second, compared to national cancer statistics at the given period of time, this study has fever stage I, more stage II and similar numbers of stage III cases [45]. As the distribution is similar to other countries without a national screening programme [26], and is not skewed heavily towards early or late stage cancers, the results are generalizable to other comparable countries. Selection bias may be present due to differences between participants and nonparticipants in the HUNT study [46]. We had not enough information about other factors possibly influencing survival, such as comorbidities or the cause of death, making cancer specific survival analysis or competing risk analysis possible. This introduces the possibility for bias as the differences in survival could potentially be due to other factors than CRC. The influence of other cancer diagnoses on overall survival or the ctDNA markers present could not be ruled out, but as the investigated ctDNA markers are established diagnostic markers of CRC, an origin in other tumour tissue is less likely. In a pre-diagnostic or screening setting, too many exclusion criteria could hamper clinical translation of the test. Additionally, as the sample size was relatively small, the number of covariates and subgroup analyses had to be chosen cautiously.

One strength of this study is the quality of the plasma samples in HUNT Biobank and the large number of methylated markers examined. Although the plasma samples were prepared 11–13 years prior to analysis and not explicitly handled or stored for such analyses, ctDNA was detectable. Studies have shown that the amounts of ctDNA in stored plasma samples may decrease over time [47]. Additionally, the amount of plasma used for this study was small (2 × 900 μl). In real life these samples would be fresh, handled explicitly for this analysis and of larger quanta, probably making ctDNA easier to detect. Additionally, most other studies have collected blood samples at time of diagnosis, making the potential biomarkers prone to be influenced by cancer-related lifestyle changes, treatments or interventions around the time of diagnosis. This is an advantage of the prediagnostic timing of sample collection. Second, the survival status and clinicopathological data are very accurate due to thorough examination of the medical records.

Methylation specific PCR of plasma samples represents an attractive method for predicting prognosis in CRC. Several studies have shown promising results using methylated ctDNA as a biomarker after primary treatment. This study expands the use of these biomarkers to predict prognosis up to two years prior to clinical diagnosis, challenging the position of CEA and CT as the prognostic tools guiding treatment today. Possible clinical impacts could be neoadjuvant treatment or more comprehensive surgeries for those in the groups with worse prognosis who tolerate such approaches. Additionally, those with a worse prognosis who do not tolerate these treatments may be spared unnecessary side-effects. Conversely, patients in the group with better prognosis could avoid comprehensive treatments or be offered minimally invasive treatment despite frail health.

To address the generalisability of these findings, the ctDNA markers should be externally validated in other cohorts and are now considered ready for phase 3 studies in larger cohort in studies designed for this purpose and with suitable controls in an early diagnosis/screening-like setting.

## Conclusions

This study presents ctDNA marker panels as a prognostic tool for CRC. These panels of markers could offer a more accurate prognosis prediction at the time of diagnosis and consequently guide more appropriate treatment choices for future patients. To externally validate our findings, prospective clinical studies should be performed at the time of clinical diagnosis or possibly in the screening setting.

## Supplementary Information

Supplementary Material 1.

## Data Availability

The dataset supporting the conclusions of this article is available from a third party and is not publicly available. The data that support the findings of this study are deidentified participant data as well as biological materials, available from HUNT upon application (https://www.ntnu.no/hunt, e-mail: kontakt@hunt.ntnu.no). Restrictions apply to the availability of these data, which were used under licence for this study. The authors declare no competing interests.

## Abbreviations

AJCC: American Joint Committee on Cancer
BMI: Body Mass Index
CEA: Carcinoembryonic antigen
CRC: Colorectal cancer
CRN: The Cancer Registry of Norway
ctDNA: Circulating tumour DNA
DNA: Deoxyribonucleic acid
EDTA: Ethylenediaminetetraacetic acid
EMVI: Extramural vascular invasion
GRIA4: Glutamate ionotropic receptor AMPA type subunit 4
Hb: Haemoglobin
HER2: Human epidermal growth factor receptor 2
HR: Hazard ratio
HUNT: The Trøndelag Health Study
HUNT-CRC_OS_: The Trøndelag Health Study colorectal cancer overall survival panel (GRIA4, RARB, SLC8A1, VIM and WNT5A)
HUNT-CRC_RFS_: The Trøndelag Health Study colorectal cancer recurrence-free survival panel (RARB, SDC2, SLC8A1 and WNT5A)
HUNT-CRC_PP_: The Trøndelag Health Study colorectal cancer poor prognosis panel (RARB, SDC2 and WNT5A)
LVI: Lymphovascular invasion
MMR: Mismatch repair
MSI: Microsatellite instability
NTRK: Neurotrophic tyrosine receptor kinase
OS: Overall survival
PNI: Perineural invasion
PP: Poor prognosis
RARB: Retinoic acid receptor beta
RFS: Recurrence-free survival
SDC2: Syndecan 2
SLC8A1: Solute carrier family 8 member A1
TNM: Tumour, Node, Metastasis
VIM: Vimentin
WNT5A: Wnt family member 5A
