# Supplementary material for: The prognostic role of circulating tumour DNA detected prior to clinical diagnosis of colorectal cancer in the HUNT study
Source: BMC Cancer. 2024 Oct 10;24:1251. doi: 10.1186/s12885-024-13030-x (PMC11465842; doi:10.1186/s12885-024-13030-x)
Supplement: Supplementary file 1 — Supplementary Material 1. [file 12885_2024_13030_MOESM1_ESM.docx]

**The prognostic role of circulating tumour DNA detected prior to clinical diagnosis of colorectal cancer in the HUNT study**

Siv S. Brenne^1, 2^, Poul H. Madsen^3, 4^, Inge S. Pedersen^3, 4, 5^, Kristian Hveem^2^, Frank Skorpen^6^, Henrik B. Krarup^3, 4, 5^, Athanasios Xanthoulis^1,6^, Eivor A. Laugsand^1, 2^

^1^ Department of Surgery, Levanger Hospital, Nord-Trøndelag Hospital Trust, Norway

^2^ HUNT Research Centre, Department of Public Health and Nursing, Norwegian University of Science and Technology, Levanger, Norway

^3^ Clinical Cancer Research Centre, Aalborg University Hospital, Aalborg, Denmark

^4^ Molecular Diagnostics, Aalborg University Hospital, Aalborg, Denmark

^5^ Department of Clinical Medicine, Aalborg University, Aalborg, Denmark

^6^ Department of Clinical and Molecular Medicine, Norwegian University of Science and Technology, NTNU, N-7489 Trondheim, Norway

**Correspondence:** Siv Stakset Brenne, M.D., HUNT Research Centre, Department of Public Health and Nursing, NTNU, Norwegian University of Science and Technology, Forskningsveien 2, N-7600 Levanger, Norway. Telephone: +47 74075180, Fax: +47 74075181, E-mail: siv.s.brenne@ntnu.no

**Supplementary Materials - Index**

| **Supplementary Methods** |  |
| --- | --- |
| Table S1 First round of PCR | *page 3* |
| Table S2 Second round of PCR | *page 5* |
| **Supplementary Figures and Tables** |  |
| Table S3 Cox regression OS, RFS and PP Stage I-III  Table S4 Comparison between groups OS, RFS and PP Stage I-III | *page 9*  *page 10* |

Table S5 Other cancers  *page 11*

**Supplementary Methods**

As the method was not quantitative, there were no reference intervals, ranges or CVs (coefficient of variation). Units of measurement were cycle threshold (Ct) values· *MEST1* was used as a quality control parameter (naturally hemimethylated reference gene), to ensure the correct assessment of the other methylation markers. The corrected Ct value was used for analyses. The correction factor accounted for differences in PCR effectivity in different promoter sequences and was calculated as the mean Ct value of eight replicates of global methylated DNA (EpiTect methylated human control DNA) minus the mean Ct of *MEST1.* All samples with Ct values ≤ 40 were deemed positive (methylated). The remaining were deemed negative (unmethylated). For gene markers *BCAT1*, *GRIA4*, *SDC2*, *SEPT9, SFRP2, TWIST1, VIM* and *WNT5A* sense promoter sequences were used. For *FLI1*, *IKZF1, NPTX2, RARB* and *SLC8A1* antisense promoter sequences were used (Table S3).

First round of PCR

To amplify the amount of deaminated DNA of interest, a first round of PCR was conducted using a mix of outer methylation-specific primers for all promoter regions tested. The reaction buffer for each sample consisted of 25 μl containing PCR stock, 13 μM MgCl2, 0·6 mM dNTP, 250 nM of each outer primer and 1·5 U Taq polymerase (Bioline® [Taunton, MA, USA]). Twenty-five microliters of purified deamination product were added to each tube containing the first round reaction mix. PCR was performed for 20 cycles at 92 °C for 15 s, 55 °C for 30 s and 72 °C for 30 s.

Second round of PCR

Ten microliters of mix containing 0·4 μM inner methylation-specific primers and methylation-specific probes were distributed in 30 individual wells in a 96-well PCR plate. Ten microliters of first round PCR product were added to 710 μl of reaction mix containing PCR stock, 250 μM dNTP, 10 μM MgCl2, and 15 U Taq polymerase (Bioline® [Taunton, MA, USA]). Twenty microliters of the reaction mix were added to each of the 30 wells containing primers and probes. Real-time PCR was carried out for 45 cycles at 94 °C for 15 s, 55 °C for 30 s (annealing and detection) and 72 °C for 30 s.

**Table S1 First round of PCR**

| **Associated gene** | **NCBI Ref· Sequence**  (nucleotide position) | **Amplicon size** (bp) |
| --- | --- | --- |
| ***AGBL4*** | **NC_000001·11** |  |
| *AGBL4 AoF* | TTTTGTGTTTTAGGGGTGGC (50024052-50024218) | 169 |
| *AGBL4 AoR* | CGCGACTAAAAACGAAAAAAACG (50024198-50024221) |  |
| *AGBL4 BoF* | TTACGAAAAAACAACGCCCCG (50024042-50024062) | 109 |
| *AGBL4 BoR* | TTGAGGGCGGGAGGGAC (50023953-50023969) |  |
| ***ALX4*** | **NC_000011·10** |  |
| *ALX4 AoF* | GTCGGGAGGGTTCGTC (44309968-44309983) | 114 |
| *ALX4 AoR* | CGAACCCGACTCTTAACG (44309869-44309886) |  |
| *ALX4 BoF* | CGATTTAAAAAAAACTACTCTCG (44311642-44311664) | 201 |
| *ALX4 BoR* | GGAGGTTTAGAGAGGGGC (44311463-44311480) |  |
| ***BCAT1*** | **NC_000012·12** |  |
| *BCAT1 AoF* | GTAATTTAGTTCGTTACGTGTATTC (24949081-24949101) | 120 |
| *BCAT1 AoR* | CAAAAATCGTAACCCCTAACCG (24948981-24949002) |  |
| *BCAT1 BoF* | CGATCTACAATCCAACCCG (24949089-24949107) | 126 |
| *BCAT1 BoR* | TAAGGGTCGTAGTTTTTGGTC (24948981-24949000) |  |
| ***BMP3*** | **NC_000004·12** |  |
| *BMP3 AoF* | TAGCGTTGGAGTGGAGAC (81031015-81031032) | 114 |
| *BMP3 AoR* | CCAACCCCACTTACTACG (81031112-81031129) |  |
| *BMP3 BoF* | AAATCTCTAAACACATACTACG (81031448-81031469) | 101 |
| *BMP3 BoR* | GAGGGCGTTAGGGTTGC (81031533-81031549) |  |
| ***FLI1*** | **NC_000011·10** |  |
| *FLI1 AoF* | GTAGGGAGGGTTTAGGGC (128694139-128694156) | 93 |
| *FLI1 AoR* | CAATATTCCACACATTAACCCG (128694211-128694232) |  |
| *FLI1 BoF* | AACTCCCGTAACCCCAACG (128694644-128694662) | 100 |
| *FLI1 BoR* | TAGGGTTTATTAGGTAGGGATTC (128694726-128694744) |  |
| ***GRIA4*** | **NC_000011·10** |  |
| *GRIA4 AoF* | GGGTGAAGTTAGTCGAGC (105610442-105610459) | 132 |
| *GRIA4 AoR* | CTCCAAACTCGCATCAACCG (105610556-105610574) |  |
| *GRIA4 BoF* | CCAAAAAAACAACGATACTCCG (105610509-105610528) | 125 |
| *GRIA4 BoR* | TTGTTTTATRCGTATTCGCGTTC (105610631-105610653) |  |
| ***IKZF1*** | **NC_000007·14** |  |
| *IKZF AoF* | GGTATTCGTATTTTGGGGTC (50303622-50303641) | 153 |
| *IKZF AoR* | GCTCGCCCCGCTAACG (50303759-50303775) |  |
| *IKZF BoF* | TACGTCACCCCAAAATTTACG (50304413-50304433) | 139 |
| *IKZF BoR* | GCGTCGTTCGCGGGTTTC (50304535-50304552) |  |
| ***NDRG4*** | **NC_000016·10** |  |
| *NDRG4 AoF* | GGTGTTTTTTAGGTTTCGC (58463468-58463486) | 144 |
| *NDRG4 AoR* | ACCCGCGAACGATACCG (58463595-58463612) |  |
| *NDRG4 BoF* | ACCGACCGCCACTTTCCG (58464489-58464506) | 90 |
| *NDRG4 BoR* | GGTTCGTATTTTTTTTTGTTTAGATTC (58464553-58464579) |  |
| ***NPTX2*** | **NC_000007·14** |  |
| *NPTX2 AoF* | GGCGTTCGTTCGTTTATGTC (98617416-98617435) | 128 |
| *NPTX2 AoR* | ACGAAACGACTACCGAACG (98617525-98617544) |  |
| *NPTX2 BoF* | GCGAACGCCAAATACCCG (98618200-98618218) | 113 |
| *NPTX2 BoR* | CGCGGTAGTTTCGTAGTATTC (98618293-98618313) |  |
| ***PRIMA1*** | **NC_000014·9** |  |
| *PRIMA1 AoF* | GAGTTCGGGTATTTTAGTGTTTC (93788903-93788927) | 161 |
| *PRIMA1 AoR* | CAAAACCAAAACTAACCGCCG (93788766-93788786) |  |
| *PRIMA1 BoF* | AAATACCTACGCGATATCCG (93787778-93787797) | 144 |
| *PRIMA1 BoR* | CGCGTAGTGTAGTAGTAGC (93787653-93787671) |  |
| ***RARB*** | **NC_000003·12** |  |
| *RARB AoF* | AGTAGGGTTTGTTTGGGTATC (25428388-25428408) | 127 |
| *RARB AoR* | TCGACCAATCCAACCGAATCG (25428495-25428515) |  |
| *RARB BoF* | AAATATAAACTAAAAAACGAAACG (25427861-25427884) | 159 |
| *RARB BoR* | AATGAGTAGGGGAGGAGTC (25428002-25428020) |  |
| ***SEPT9*** | **NC_000017·11** |  |
| *SEPT9 AoF* | GTTTAGTATTTATTTTCGAAGTTC (77373542-77373560) | 116 |
| *SEPT9 AoR* | GCC GAA AAC GCT TCC TCG (77373444-277373459) |  |
| *SEPT9 BoF* | CGAAAACTCGCGAAACTACCTCG (77373872-77373894) | 169 |
| *SEPT9 BoR* | AGGACGCGGTTGTTTATTTAGT (77374022-77374041) |  |
| ***SDC2*** | **NC_000008·11** |  |
| *SDC2 AoF* | GCGTAGGAGGAGGAAGC (96494093-96494109) | 102 |
| *SDC2 AoR* | CAACCCGCGCACACG (96494181-96494195) |  |
| *SDC2 BoF* | CAATCCCCAAATATACACCG (96494429-96494448) | 155 |
| *SDC2 BoR* | GCGGTTAGATTAAATCGTAATTTTC (96494560-96494584) |  |
| ***SFRP1*** | **NC_000008·11** |  |
| *SFRP1 T AoF* | GGTATAGTCGTAGGTTCGC (41038955-41038973) | 127 |
| *SFRP1 T AoR* | AACCGTAAACTCGACCAACG (41309063-41309082) |  |
| *SFRP1 BoF* | GACTCCGAAAACCACAACG (41308335-41308353) | 116 |
| *SFRP1 BoR* | CGTTTGGTTTGTTTTAGGTATC (41308434-41308451) |  |
| ***SFRP2*** | **NC_000004·12** |  |
| *SFRP2 AoF* | GTTTTTCGGAGTTGCGCGC (153789028-153789046) | 124 |
| *SFRP2 AoR* | CTCTTCGCTAAATACGACTCG (153788922-153788942) |  |
| *SFRP2 BoF* | CGTTATCCTAAAAAAAACGATCG (153788388-153788410) | 145 |
| *SFRP2 BoR* | CGTTTGTTTCGATGATTTAGAC (153788512-153788533) |  |
| ***SLC8A1*** | **NC_000002·12** |  |
| *SLC8A1 AoF* | TGATGTGGTGTGTGCGC (40451723-40451739) | 180 |
| *SLC8A1 AoR* | ATCCTCAAAACCCAACGCG (40451559-40451577) |  |
| *SLC8A1 BoF* | GCTACCCGCTACCGCG (40452085-40452100) | 116 |
| *SLC8A1 BoR* | AGTTTTCGATTAGGAAGAGGT (40451984-40452005) |  |
| ***TWIST1*** | **NC_000007·14** |  |
| *TWIST1 AoF* | GAAGTTTGCGGGTTGTGGC (19117076-19117094) | 129 |
| *TWIST1 AoR* | CTAACGCTCCCGCAGC (19116965-19116980) |  |
| *TWIST1 BoF* | CGAAAAACTACAAACGCAACG (19116993-19117013) | 98 |
| *TWIST1 BoR* | GGGATGATTTTTCGTAGCGC (19116915-19116934) |  |
| ***VIM*** | **NC_000010·11** |  |
| *VIM AoF* | GAGGTTTTCGCGTTAGAGAC (17229296-17229315) | 143 |
| *VIM AoR* | ACGAACCTAATAAACATAACTACG (17229416-17229439) |  |
| *VIM BoF* | AAACTTAAATCAATCTAATCTAACG (17228450-17228474) | 103 |
| *VIM BoR* | GGGTCGTTTAGTTATCGGC (17228535-17228553) |  |
| ***WNT5A*** | **NC_000003·12** |  |
| *WNT5A AoF* | CGTGGAATAGTTGTTTGC (55487294-55487311) | 152 |
| *WNT5A AoR* | CGAACCTAAACTCCCG (55487159-55487174) |  |
| *WNT5A BoF* | AAAACGAAACGCCTCTCCG (55487310-55487329) | 85 |
| *WNT5A BoR* | TAGGGTTTGGTCGGGGC (55487244-55487260) |  |
| ***ZNF331*** | **NC_000019·10** |  |
| *ZNF331 AoF* | GACGGGTTTTCGTGGTTTAC (53554924-53554943) | 114 |
| *ZNF331 AoR* | CTAACCTAAAACACATCTACG (53555018-53555038) |  |
| *ZNF331 BoF* | TAATAAAAACCGTACTCGATACG (53554624-53554646) | 124 |
| *ZNF331 BoR* | TATCGTTAGAAGTTTCGTGTTC (53554727-53554748) |  |
| ***MEST1v1*** | **NC_000007·14** |  |
| *MEST1v1 Ac* | GGTTTTAAAAGTCGGTGTTTATT (130492052-130492074) | 130 |
| *MEST1v1 At* | GGTTTTAAAAGTTGGTGTTTATT (130492052-130492074) |  |
| *MEST1v1 B** | CCIAACAACTACAACCACTCC (130492162-130492182) |  |

Primers used for first round amplification. AoF, AoR and BoF, BoF correspond to outer forward and reverse primers for sense and antisense promoter sequences, respectively.

MEST1v1: Ac forward primer for methylated MEST1v1, At forward primer for unmethylated MEST1v1. Primer B is reverse primer for both methylated and unmethylated MEST1v1. *I, inosine

**Table S2 Second round of PC****R**

| **Associated gene** | **NCBI Ref· Sequence**  (nucleotide position) | **Amplicon size** (bp) |
| --- | --- | --- |
| ***AGBL4*** | **NC_000001·11** |  |
| *AGBL4 AF* | GAGTTTGTTTAGTTGTTAGGATGC (50024073-50024096) | 148  148 |
| *AGBL4 AR* | CGCGACTAAAAACGAAAAAAACG (50024198-50024221) |  |
| *AGBL4 A probe* | HEX-CGAAACGCTACTCCTCCGCAACCG-BHQ-1 (50024104-50024128) |  |
| *AGBL4 BF* | CAAATCCCAACTCAAACCCG (50024017-50024036) | 83  83 |
| *AGBL4 BR* | TTGAGGGCGGGAGGGAC (50023953-50023969) |  |
| *AGBL4 B probe* | FAM-CGAAAACGAAACCTTAAACGCACGCTCCT-BHQ-1 (50023977-50024005) |  |
| ***ALX4*** | **NC_000011·10** |  |
| *ALX4 AF* | TTTTTCGGAGGCGATAAGTTC (44309954-44309934) | 85  85 |
| *ALX4 AR* | CGAACCCGACTCTTAACG (44309869-44309886) |  |
| *ALX4 A beacon* | FAM-CGCGATTGTCGGTCGTCGTTAAAGTATCGCG-dabcyl (44309901-44309921) |  |
| *ALX4 BF* | CAACTTAAACCATTCCACCG (44311608-44311630) | 134  134 |
| *ALX4 BR* | GTTATAGTTGGGAAATAGTCGTC (44311496-44311520) |  |
| *ALX4 B probe* | HEX-CCTCTACATCTACGCTATCCGCACGAC-BHQ1 (44311568-44311594) |  |
| ***BCAT1*** | **NC_000012·12** |  |
| *BCAT1 AF* | CGTGTATTCGTCGTCGTTTC (24949066-24949085) | 93  93 |
| *BCAT1 AR* | ACCCCTAACCGTATAAACCG (24948992-24949011) |  |
| *BCAT1 A probe* | FAM-TACGACTACAAAACGCGATCCCGACTACA-BHQ1 (24949016-24949044) |  |
| *BCAT1 BF* | ATACTCGCCGCCGCCTCG (24949065-24949082) | 101  101 |
| *BCAT BR* | TAAGGGTCGTAGTTTTTGGTC (24948981-24949000) |  |
| *BCAT1 B probe* | HEX-AACCGAAACCGCGCTCTACAACCG-BHQ1 (24949018-24949041) |  |
| ***BMP3*** | **NC_000004·12** |  |
| *BMP3 AF* | AGTGGAGACGGCGTTC (81031024-81031039) | 96  96 |
| *BMP3 AR* | CTTACTACGCTAACCCAACG (81031101-81031120) |  |
| *BMP3 A beacon* | FAM-CGTCGAGCGGGTGAGGTTCGCGTATCGACG-dabcyl (81031052-81031069) |  |
| *BMP3 BF* | CTCTATAACAAATACAACACG (81031471-81031491) | 78  78 |
| *BMP3 BR* | GAGGGCGTTAGGGTTGC (81031533-81031549) |  |
| *BMP3 B beacon* | HEX-CGCGATCAAACGACCCGAACACCGAGATCGCG-dabcyl (81031496-81031513) |  |
| ***FLI1*** | **NC_000011·10** |  |
| *FLI1 AF* | GTAGGGAGGGTTTAGGGC (128694139-128694156) | 74  74 |
| *FLI1 AR* | CCGATTACAACCTAACCTCG (128694194-128694213) |  |
| *FLI1 A probe* | HEX-CGAATTAACCCGACGCGACCTCCCTAA-BHQ-1 (128694158-128694184) |  |
| *FLI1 BF* | CTAAACACTAAACTTCCTCTCCG (128694663-128694685) | 77  77 |
| *FLI1 BR* | TAGGGATTCGAGTTCGGGC (128694725-128694740) |  |
| *FLI1 B probe* | FAM-AAACGAAACACGACGAAAACGAAAACTACA-BHQ-1 (128694689-128694718) |  |
| ***GRIA4*** | **NC_000011·10** |  |
| *GRIA4 AF* | CGTGCGGAGGGAGTGC (105610473-105610488) | 73  73 |
| *GRIA4 AR* | AACAAACCTACACCAACCCG (105610527-105610546) |  |
| *GRIA4 A probe* | HEX-CGCTACCCTCCTAACGTCCCCTCCTCG-BHQ-1 (105610495-105610521) |  |
| *GRIA4 BF* | CGAACTAATACGAACTAAAAAACG (105610556-105610578) | 97  97 |
| *GRIA4 BR* | TTGTTTTATTCGTATTCGCGTTC (105610631-105610653) |  |
| *GRIA4 B probe* | FAM-CGTATAACGACCGCGACG-BHQ-1 (105610580-105610596) |  |
| ***IKZF1*** | **NC_000007·14** |  |
| *IKZF1 AF* | TGGGGTCGGAGTTTGAATTC (50303635-50303654) | 124  124 |
| *IKZF1 AR* | GAAACTAAAAAAACTCGAAACATCG (50303736-50303759) |  |
| *IKFZ1 A probe* | HEX-CGCCCGCTATACGCTCAACTTCACG-BHQ-1 (50303683-50303707) |  |
| *IKZF1 BF* | TACGTCACCCCAAAATTTACG (50304413-50304433) | 103  103 |
| *IkZF1 BR* | GTCGGTCGTTTTCGTCGC (50304499-50304516) |  |
| *IKZF1 B probe* | FAM-CGCTCTAACCACCCGCCGCTCTAAACG-BHQ-1 (50304449-50304475) |  |
| ***NDRG4*** | **NC_000016·10** |  |
| *NDRG4 AF* | GTTCGTTTATCGGGTATTTTAGTC (58463515-58463534) | 97  97 |
| *NDRG4 AR* | ACCCGCGAAACGATACCG (58463595-58463612) |  |
| *NDRG4 A beacon* | HEX-CGCGATCGCGGTTCGTTCGGGATTAGTTGATCGCG-dabcyl (58463567-58463587) |  |
| *NDRG4 BF* | GCCACTTTCCGAATTAAAACG (58464496-58464516) | 83  83 |
| *NDRG4 BR* | GGTTCGTATTTTTTTTTGTTTAGATTC (58464553-58464579) |  |
| *NDRG4 B probe* | FAM-CGAACGCGACGACCGAAAACTAAAACG-BHQ-1 (58464522-58464548) |  |
| ***NPTX2*** | **NC_000007·14** |  |
| *NPTX2 AF* | GGCGTTCGTTCGTTTATGTC (98617416-98617435) | 101  101 |
| *NPTX2 AR* | GAACTATCCTAAACCCCAACG (98617497-98617517) |  |
| *NPTX2 A probe* | HEX-CGCCACGCTAACGACCAACAACGC-BHQ-1 (98617468-98617488) |  |
| *NPTX2 BF* | CCAAATACCCGAAAACGCG (98618208-98618226) | 105  105 |
| *NPTX2 BR* | CGCGGTAGTTTCGTAGTATTC (98618293-98618313) |  |
| *NPTX2 B probe* | FAM-CGAACCGAAATACGCTCCCGCAAACC-BHQ-1 (98618263-98618288) |  |
| ***PRIMA1*** | **NC_000014·9** |  |
| *PRIMA1 AF* | GGGAGTTAACGCGGGTTC (93788854-93788871) | 88  88 |
| *PRIMA1 AR* | GCCGAAACGAAAACAAAAAACG (93788783-93788804) |  |
| *PRIMA1 A probe* | HEX-AAATCTCCTCCACGCCGCCTAAAACCG-BHQ-1 (93788809-93788835) |  |
| *PRIMA1 BF* | CCTTACTCTCCTAACAATTCG (93787746-93787766) | 96  96 |
| *PRIMA1 BR* | GCGAGCATTAGTAGTAGTTAC (93787670-93787690) |  |
| *PRIMA1 B probe* | FAM-CGAACGCCGCTAACCGAAATACTCCTC-BHQ-1 (93787710-93787736) |  |
| ***RARB*** | **NC_000003·12** |  |
| *RARB AF* | GGGTTTGTTTGGGTAATCGTC (25428392-25428411) | 123  123 |
| *RARB AR* | TCGACCAATCCAACCGAAACG (25428495-25428515) |  |
| *RARB A beacon* | HEX-CGCGACGAA+TA+GTT+CGAATCGCG-dabcyl (25428421-25428435) |  |
| *RARB BF* | CTAAAAAACGAAACGATAAACG (25427870-25427891) | 69  69 |
| *RARB BR* | GGCGGTTTAGTTTGGAAAAC (25427920-25427939) |  |
| *RARB B probe* | FAM-ACGAACGAACGCAAACGAAACACCG-BHQ-1 (25427896-25427920) |  |
| ***SEPT9*** | **NC_000017·11** |  |
| *SEPT9 AF* | GTTTAGTATTTATTTTCGAAGTTC (77373542**-**77373560) | 93  93 |
| *SEPT9 AR* | CCTCCGCGCGACCCG (77373467**-**77373481) |  |
| *SEPT9 A beacon*  *(FAM)* | FAM-CGACGTATTTAGTTGCGCGTTGATCGACGTCG-dabcyl (77373511- 77373530) |  |
| *SEPT9 BF* | CACAATTTCACTCTAAAAAATCCATCG (77373910-77373936) | 101  101 |
| *SEPT9 BR* | GGACGCGGTTGTTTATTTAGTC (77373992-77374011) |  |
| *SEPT9 B probe* | HEX-CCCGACGACTCTCGAACCCCGCTTA-BHQ-1 (77373657-77373681) |  |
| ***SDC2*** | **NC_000008·11** |  |
| *SDC2 AF* | GTTTCGAGTTCGAGTTTTC (96494122-96494140) | 66  66 |
| *SDC2 AR* | CGCACACGAATCCG (96494175-96494188) |  |
| *SDS2 A beacon*  *** | FAM-CGCGATCCGTGAGT+GTAAT+GTTG+GGTGATCGCG-dabcyl (96494146-96494165) |  |
| *SDC2 BF* | CCAAATATACACCGAAAATCCG (96494435-96494456) | 105  105 |
| *SDC2 BR* | ATCGCGGGTAACGTTAGC (96494522-96494540) |  |
| *SDC2 B probe* | HEX-AAACAAATACGCTCGTCCGATCACCCTTT-BHQ-1 (96494460-96494488) |  |
| ***SFRP1*** | **NC_000008·11** |  |
| *SFRP1 AF* | GGTATAGTCGTAGGTTCGC (41038955-41038973) | 115  115 |
| *SFRP1 AR* | GACCAACGAATACGACTACG (41309051-41309070) |  |
| *SFRP1 A probe* | HEX-CGAACATCGACCCGTACCAAAACGAACG-BHQ-1 (41309011-41309038) |  |
| *SFRP1 BF* | ACCACAACGAATCCCTACG (41308345-41308363) | 93  93 |
| *SFRP1 BR* | GTATCGTTTAGCGTTTTTCGC (41308418-41308438) |  |
| *SFRP1 B probe* | FAM-CGCAAAACTACCGCTCCCGAACCAA-BHQ-1 (41308373-41308397) |  |
| ***SFRP2*** | **NC_000004·12** |  |
| *SFRP2 AF* | GTTTTTCGGAGTTGCGCGC (153789028-153789046) | 98  98 |
| *SFRP2 AR* | CCGAAAAACTAACAACCGACG (153788948-153788968) |  |
| *SFRP2 A beacon* | HEX-CGACGTTTGTAGCGTTTCGTTCGCGTTGTTACGTCG-dabcyl) (153789000-153789023) |  |
| *SFRP2 BF* | GATCGCACTCAAACATATCG (153788406-153788425) | 107  107 |
| *SFRP2 BR* | ACGAGATTATTTAGTTATGTTATTC (153788490-153788513) |  |
| *SFRP2 B probe* | FAM-AAACGAACATAACCGAAACGCAACGATCC-BHQ-1 (153788442-153788470) |  |
| ***SLC8A1*** | **NC_000002·12** |  |
| *SLC8A1 AF* | TGATGTGGTGTGTGCGC (40451723-40451739) | 136  136 |
| *SLC8A1 AR* | ACAACCCTTTCTTCCTCCG (40451603-40451621) |  |
| *SLC8A1 A probe* | HEX-CACGCCCAAACGCACCAACCCGAA-BHQ-1 (40451654-40451677) |  |
| *SLC8A1 BF* | GCTACCCGCTACCGCG (40452085-40452100) | 84  84 |
| *SLC8A1 BR* | TTTGTGGGAAGTATAAAGTAGGC (40452016-40452038) |  |
| *SLC8A1 B probe* | FAM-TCGCGCCGACGCTACACGAT-BHQ-1 (40452057-40452076) |  |
| ***TWIST1*** | **NC_000007·14** |  |
| *TWIST1 AF* | GTAGTTCGGTTTAGGGTAAGC (19117102-19117122) | 157  157 |
| *TWIST1 AR* | CTAACGCTCCCGCACG (19116965-19116980) |  |
| *TWIST1 A probe* | HEX-CGAACTCCCGCCGCCGCTACTACTA-BHQ-1 (19117022-19117046) |  |
| *TWIST1 BF* | CGCAACGAATCATAACCAACG (19116979-19117000) | 85  85 |
| *TWIST1 BR* | GGGATGATTTTTCGTAGCGC (19116915-19116934) |  |
| *TWIST1 B probe* | FAM-CGCCAACGCACCCAATCGCTAAACG-BHQ-1 (19116946-19116970) |  |
| ***VIM*** | **NC_000010·11** |  |
| *VIM AF* | ATATTTATCGCGTTTTCGTTC (17229337-17229357) | 102  102 |
| *VIM AR* | ACGAACCTAATAAACATAACTACG (17229416-17229439) |  |
| *VIM A beacon* | FAM-CGACGTGTTCGCGTTATCGTCGTCGACGTCG-dabcyl (17229377-17229395) |  |
| *VIM BF* | CTAACGATTTCCCCTAAACCG (17228469-17228489) | 84  84 |
| *VIM BR* | GGGTCGTTTAGTTATCGGC (17228535-17228553) |  |
| *VIM B probe* | HEX-ACCCTCAATCGACGAAACAACAAAACGCG-BHQ-1 (17228496-17228524) |  |
| ***WNT5A*** | **NC_000003·12** |  |
| *WNT5A AF* | CGTGGAATAGTTGTTTGC (55487294-55487311) | 134  134 |
| *WNT5A AR* | TTAAAACAAAACTAAAATACG (55487177-55487197) |  |
| *WNT5A A beacon*  *** | HEX-CGCGATCAACCTAATC+GAAAC+GCAACTAAAGATCGCG-dabcyl (55487247-55487269) |  |
| *WNT5A BF* | TAGGGTTTGGTCGGGGC (55487244-55487260) | 68  68 |
| *WNT5A BR* | CCGTAAAACAATTACCTACGCG (55487292-55487312) |  |
| *WNT5A B probe* | FAM-CGCCGAACCGACGACTCCCTAATT-BHQ-1 (55487263-55487286) |  |
| ***ZNF331*** | **NC_000019·10** |  |
| *ZNF331 AF* | TTTGTATTGTGTGGAGTGGC (53554953-53554972) | 85  85 |
| *ZNF331 AR* | CTAACCTAAAACACATCTACG (53555018-53555038) |  |
| *ZNF331 A probe* | FAM-CTAACGCCCAACACGACCACGCTAC-BHQ-1 (53554980-53555500) |  |
| *ZNF331 BF* | TCGATACGCTTCTACACCG (53554639-53554657) | 74  74 |
| *ZNF331 BR* | GTTATGGGCGTTAATTTTATTTGC (53554690-53554713) |  |
| *ZNF331 B probe* | HEX-CGCAACCGCTATATCTCCGCCAATCCG-BHQ-1 (53554662-53554688) |  |
| ***MEST1v1 ***** | **NC_000007·14** |  |
| *MEST1v1 MF* | CGCGGTAATTAGTATATTTC (130492085-130492107) | 74  74 |
| *MEST1v1 MR* | GCTACGACACTACGCTTACG (130492135-130492159) |  |
| *MEST1v1 M beacon* | HEX-CGCGATCGGTAGTTGCGTTTCGTATCGCG-dabcyl (130492121-130492137) |  |
| *Mest1V1 UF* | GTTGTTGTGGTAATTAGTATATTTT (130492083-130492107) | 78  78 |
| *MEST1v1 UR* | AACATACACTACAACACTACA (130492141-130492161) |  |
| *MEST1v1 U beacon* | FAM-CGCGATGTGGTAGTTGTGTTTTGTCGCG-dabcyl (130492119-130492136) |  |

Methylation specific primers and probes used for RT-PCR. AF, AR and BF, BF correspond to forward and reverse primers for sense and antisense promoter sequences, respectively.

*(+) denotes LNA nucleosides.

**MEST1v1: MF and MR, forward and reverse primers for methylated MEST1v1. MEST1v1 M beacon, methylation specific probe. UF and UR, forward and reverse primers for unmethylated MEST1v1. MEST1v1 U beacon, unmethylation specific probe.

**Supplementary Figures and Tables**

**Table S3 Cox regression OS, RFS and PP Stage I-III**

|  | OS (adj for age) | | | RFS (adj for age) | | | PP (adj for age) | | | Methylation  % |
| --- | --- | --- | --- | --- | --- | --- | --- | --- | --- | --- |
| *Variables for inclusion* | HR | 95% CI | p-value | HR | 95% CI | p-value | HR | 95% CI | p-value |  |
| *AGBL4 B* | 1.289 | 0.614-2.705 | 0.503 | 1.498 | 0.415-5.412 | 0.537 | 1.508 | 0.565-4.026 | 0.413 | 72.9 |
| *ALX4 A* | 1.501 | 0.630-3.579 | 0.359 | 1.260 | 0.281-5.645 | 0.762 | 1.588 | 0.544-4.637 | 0.398 | 11.8 |
| *BCAT1 B* | 1.305 | 0.710-2.398 | 0.392 | 0.571 | 0.177-1.841 | 0.348 | 0.889 | 0.392-2.016 | 0.778 | 40.0 |
| *BMP3 B* | 1.925 | 0.750-4.941 | 0.173 | 2.540 | 0.553-11.658 | 0.231 | 1.116 | 0.262-4.760 | 0.882 | 7.1 |
| *FLI1 A* | 1.626 | 0.861-3.072 | 0.134 | 1.892 | 0.593-6.036 | 0.282 | 1.992 | 0.831-4.773 | 0.122 | 57.6 |
| *GRIA4 A* | 1.957 | 1.056-3.626 | **0.033** | 1.418 | 0.469-4.281 | 0.536 | 1.784 | 0.795-3.999 | 0.160 | 48.2 |
| *IKZF1 B* | 2.786 | 0.970-8.003 | 0.057 | 1.923 | 0.242-15.203 | 0.535 | 0.801 | 0.108-5.953 | 0.829 | 4.7 |
| *NDRG4 A* | 0.373 | 0.051-2.722 | 0.331 | 1.651 | 0.211-12.916 | 0.633 | 0.755 | 0.102-5.595 | 0.784 | 4.7 |
| *NPTX2 B* | 1.386 | 0.713-2.399 | 0.386 | 1.227 | 0.412-3.657 | 0.713 | 1.306 | 0.596-2.865 | 0.505 | 42.4 |
| *PRIMA B* | 1.272 | 0.603-2.682 | 0.527 | 1.490 | 0.323-6.870 | 0.609 | 2.388 | 0.710-8.037 | 0.160 | 80.0 |
| *RARB B* | 9.480 | 2.998-29.979 | **<0.001** | 9.671 | 2.540-36.813 | **<0.001** | 6.112 | 1.685-22.179 | **0.006** | 4.7 |
| *SEPT9v2 B* | 1.492 | 0.787-2.829 | 0.220 | 1.262 | 0.432-3.682 | 0.671 | 0.915 | 0.416-2.014 | 0.825 | 51.8 |
| *SDC2 A* | 1.793 | 0.876-3.670 | 0.110 | 3.383 | 1.074-10.660 | **0.037** | 2.785 | 1.195-6.490 | **0.018** | 18.8 |
| *SFRP1 A* | 1.403 | 0.762-2.584 | 0.277 | 1.198 | 0.414-3.467 | 0.739 | 1.409 | 0.641-3.094 | 0.393 | 40.0 |
| *SFRP2 A* | 1.792 | 0.954-3.365 | 0.070 | 1.632 | 0.530-5.029 | 0.394 | 1.985 | 0.885-4.452 | 0.096 | 29.4 |
| *SLC8A1 B* | 1.974 | 1.033-3.772 | **0.040** | 2.931 | 1.009-8.513 | **0.048** | 2.019 | 0.884-4.614 | 0.096 | 23.5 |
| *TWIST1 B* | 1.549 | 0.823-2.917 | 0.175 | 0.887 | 0.311-2.530 | 0.822 | 0.945 | 0.431-2.072 | 0.887 | 52.9 |
| *VIM A* | 2.948 | 1.218-7.138 | **0.017** | 3.145 | 0.661-14.967 | 0.150 | 2.921 | 0.968-8.815 | 0.057 | 7.1 |
| *WNT5A A* | 5.825 | 2.331-14.560 | **<0.001** | 6.953 | 1.812-26.677 | **0.005** | 5.574 | 3.037-15.258 | **<0.001** | 7.1 |
| *ZNF331 A* | - | - | - | - | - | - | 73556.439 | 0.000- | 0.979 | 98.8 |

A = sense, B = antisense

**Table S4 Comparison between groups OS, RFS and PP Stage I-III**

|  |  | OS |  |  | RFS |  |  | PP |  |
| --- | --- | --- | --- | --- | --- | --- | --- | --- | --- |
| Markers | 0-1 | ≥2 | p-value | 0-1 | ≥2 | p-value | 0 | ≥1 | p-value |
| n (%) | 67 (78.8) | 18 (21.2) |  | 39 (45.9) | 46 (54.1) |  | 65 (76.5) | 20 (23.5) |  |
| Sex, n (%) |  |  | 0.144 |  |  | 0.119 |  |  | 0.147 |
| *Male* | 28 (41.8) | 11 (61.1) |  | 31 (79.5) | 42 (91.3) |  | 27 (41.5) | 12 (60.0) |  |
| *Female* | 39 (58.2) | 7 (38.9) |  | 8 (20.5) | 4 (8.7) |  | 38 (58.5) | 8 (40.0) |  |
| Age, mean (SD) | 69.7 (9.9) | 71.1 (10.9) | 0.586 | 69.5 (10.2) | 73.0 (8.9) | 0.266 | 68.6 (10.3) | 74.3 (8.3) | **0.026** |
| BMI, mean (SD) | 27.3 (3.9) | 27.1 (5.4) | 0.875 | 27.3 (4.0) | 27.0 (5.5) | 0.843 | 26.8 (3.6) | 28.6 (5.7) | 0.104 |
| Smoking, mean (SD) | 13.8 (17.2) | 16.4 (13.3) | 0.555 | 13.1 (16.0) | 22.0 (17.4) | 0.080 | 11.8 (13.5) | 22.6 (21.7) | **0.009** |
| CEA, mean (SD) | 9.2 (27.8) | 6.3 (9.4) | 0.701 | 9.0 (27.2) | 6.1 (5.5) | 0.724 | 9.7 (28.7) | 5.0 (4.6) | 0.499 |
| Diabetes, n (%) | 4 (6.0) | 1 (5.6) | 0.947 | 4 (5.5) | 1 (8.3) | 0.697 | 3 (4.6) | 2 (10.0) | 0.371 |
| Other cancers, n (%) | 12 (17.9) | 5 (27.8) | 0.356 | 15 (20.5) | 2 (16.7) | 0.755 | 13 (20.0) | 4 (20.0) | 1.000 |
| Tumour localisation |  |  | 0.264 |  |  | **0.023** |  |  | 0.165 |
| *Colon* | 47 (70.1) | 15 (83.3) |  | 50 (68.5) | 12 (100.0) |  | 45 (69.2) | 17 (85.0) |  |
| *Rectum* | 20 (29.9) | 3 (16.7) |  | 23 (31.5) | 0 (0.0) |  | 20 (30.8) | 3 (15.0) |  |
| AJCC Stage, n (%) |  |  | 0.052 |  |  | **0.046** |  |  | 0.091 |
| *I* | 8 (11.9) | 0 (0.0) |  | 8 (11.0) | 0 (0.0) |  | 8 (12.3) | 0 (0.0) |  |
| *II* | 43 (64.2) | 9 (50.0) |  | 47 (64.4) | 5 (41.7) |  | 41 (63.1) | 11 (55.0) |  |
| *III* | 16 (23.9) | 9 (50.0) |  | 18 (24.7) | 7 (58.3) |  | 16 (24.6) | 9 (45.0) |  |
| Deaths, n (%) | 27 (40.3) | 15 (83.3) | **0.001** | 33 (45.2) | 9 (75.0) | 0.056 | 27 (41.5) | 15 (75.0) | **0.009** |
| Recurrences, n (%) | 9 (13.4) | 5 (27.8) | 0.145 | 8 (11.0) | 6 (50.0) | **<0.001** | 7 (10.8) | 7 (35.0) | **0.011** |
| PP events, n (%) | 16 (23.9) | 9 (50.0) | **0.031** | 17 (23.3) | 8 (66.7) | **0.002** | 13 (20.0) | 12 (60.0) | **<0.001** |
| OS, mean (SD) | 93.1 (37.5) | 64.2 (45.6) | **0.007** | 91.8 (37.8) | 57.8 (47.8) | **0.007** | 96.0 (35.1) | 57.8 (45.1) | **<0.001** |
| RFS, mean (SD) | 88.5 (42.3) | 57.3 (48.8) | **0.009** | 88.8 (41.9) | 39.8 (43.4) | **<0.001** | 92.8 (40.1) | 46.6 (43.8) | **<0.001** |
| PP, mean (SD) | 88.7 (42.0) | 57.3 (48.8) | **0.008** | 89.0 (41.7) | 39.8 (43.4) | **<0.001** | 92.8 (40.1) | 47.0 (43.4) | **<0.001** |
| HUNT3 to diagnosis, mean (SD) | 12.5 (7.1) | 11.1 (5.9) | 0.467 | 12.6 (6.9) | 9.3 (6.4) | 0.124 | 12.8 (6.8) | 10.2 (7.0) | 0.134 |

**Table S5 Other cancers**

| Organ/type | **n** |
| --- | --- |
| Myelomatosis | 1 |
| Lip | 1 |
| Mammae | 4 |
| Lung | 1 |
| Malignant melanoma | 2 |
| Prostate | 5 |
| Female genital organs | 3 |
| Skin cancer other than melanoma | 3 |
| Urinary bladder | 1 |
| Eye | 1 |
| Thyriod | 1 |
| Lymphoma | 1 |
| Pankreatic | 1 |
| Kidney | 1 |
